# Critical Limitations in Cryogenic Laser Cooling of Solids: Symmetry‐Related Fluorescence Trapping and Condensation‐Induced Parasitic Heating

**DOI:** 10.1002/advs.202519452

**Published:** 2026-01-26

**Authors:** Biao Zhong, Jiayi Zhang, Haodong Yang, Lianzhong Deng, Mauro Tonelli, Ercang Luo

**Affiliations:** ^1^ State Key Laboratory of Cryogenic Science and Technology Technical Institute of Physics and Chemistry Chinese Academy of Sciences Beijing China; ^2^ University of Chinese Academy of Sciences Beijing China; ^3^ State Key Laboratory of Precision Spectroscopy East China Normal University Shanghai China; ^4^ Dipartimento di Fisica Università di Pisa Pisa Italy

**Keywords:** anti‐Stokes fluorescence process, fluoride crystals, optical cooling, optical forces, photoluminescence

## Abstract

Laser cooling of solids to cryogenic temperatures is fundamentally limited by parasitic processes that become critically important below 100 K. While fluoride crystals like Yb^3+^‐doped LuLiF_4_ and YLiF_4_ promise cooling to below 77 K, experimental progress has stalled for a decade with the lowest temperatures plateauing in the 90–120 K range. Here, we reveal and quantify two universal, yet overlooked, limitations that dominate cryogenic laser cooling: symmetry‐dependent fluorescence trapping and condensation‐induced parasitic heating. Through combined experiment and Monte Carlo ray‐tracing on 7.5% Yb^3+^:LLF, we demonstrate that breaking the geometric symmetry of the sample is a general strategy to enhance fluorescence escape efficiency *η*_esc_ at low temperatures, thereby increasing the external quantum efficiency and lowering the global minimal achievable temperature. Furthermore, we identify water vapor condensation as the dominant parasitic heat load below 135 K, which directly absorbs pump and fluorescence radiation. Our findings establish a dual‐path strategy of geometric optimization and vacuum management that provides the critical design rules to overcome the current performance plateau. This work not only resolves a long‐standing discrepancy between theory and experiment but also delivers a universal blueprint for advancing optical refrigeration toward liquid‐nitrogen temperatures, with immediate implications for the development of vibration‐free cryocoolers in quantum technologies and space applications.

## Graphical abstract

This study uncovers two overlooked bottlenecks in cryogenic laser cooling of solids: symmetry‐driven fluorescence trapping and water vapor condensation. We demonstrate that breaking sample symmetry enhances fluorescence escape, while condensation dominates parasitic heating below 135 K. Our dual strategy provides a universal blueprint to overcome the ∼90 K plateau and paves the way toward laser cooling below 77 K.

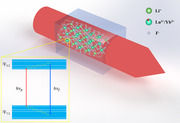

## 1 Introduction

The pursuit of all‐optical, vibration‐free cryocoolers is driven by demanding applications in space‐based infrared imaging and ultra‐stable precision metrology [1, 2, 3, 4, 5, 6, 7]. For these technologies to mature, achieving and surpassing liquid‐nitrogen temperatures (77 K) is a critical milestone. However, the field now confronts a fundamental barrier: a pervasive performance gap between theoretical predictions and experimental reality. While promising materials like Yb^3+^‐doped LuLiF_4_ (LLF) / YLiF_4_ (YLF) are predicted to reach global minimum achievable temperatures (g‐MAT) below 77 K, potentially as low as 60 K [8, 9], experimental cooling consistently plateaus significantly above this threshold, near 90–120 K [10]. This long‐standing discrepancy underscores the existence of universal, yet poorly understood, parasitic processes that dominate in the cryogenic regime. Identifying these limiting mechanisms is therefore an essential prerequisite for guiding the rational design of next‐generation optical cryocoolers and unlocking their full potential.

Meanwhile, the techniques and principles of laser cooling are being applied to an expanding range of systems. The ability to laser cool the center‐of‐mass motion of atoms, molecules, and even levitated nanoparticles to ultracold temperatures has revolutionized atomic physics over the past four decades [11, 12, 13, 14]. Now, this paradigm is extending to reduce the internal temperature of levitated nano‐ or micro‐scale particles, promising new avenues in macroscopic quantum mechanics, force sensing, and non‐equilibrium thermodynamics [15, 16, 17, 18, 19, 20, 21]. This broadening scope, which ultimately relies on efficient heat extraction from a solid object, therefore further underscores the critical need to overcome the fundamental limitations that have stalled progress in bulk systems.

The journey of optical cooling in solids dates back to P. Pringsheim in 1929 [22], culminating in its first experimental realization in 1995 with 0.3 K of net cooling in Yb^3+^‐doped ZBLAN glass [1]. Since then, laser cooling has been realized in diverse solid‐state systems, including glasses and crystals doped with various lanthanide ions (Yb^3+^, Tm^3+^, Er^3+^, Ho^3+^, and Yb^3+^/Tm^3+^ pairs), semiconductors, and nanocrystals [10, 18, 23, 24, 25, 26, 27, 28, 29, 30, 31, 32, 33, 34, 35, 36, 37, 38, 39, 40, 41]. Among lanthanides, Yb^3+^ is particularly favorable due to its simple energy level structure, absence of excited‐state absorption, and compatibility with high‐power pump lasers [42]. Consequently, the g‐MAT in solids have consistently been achieved with Yb^3+^‐doped hosts [42, 43, 44, 45]. Fluoride crystals like YLiF_4_ (YLF) and LuLiF_4_ (LLF) stand out for their exceptionally low g‐MATs, attributed to their broad transparency, low refractive index, suppressed non‐radiative relaxation, and narrow ground‐state manifolds [8, 25, 34, 46, 47, 48, 49]. The current record of 87 K was achieved using an Yb^3+^/Tm^3+^ co‐doped YLF crystal [8, 34].

Despite these advances, cooling performance plateaus around 90–123 K, deviating significantly from predictions for nearly a decade [10]. This limitation has been linked to a critical reduction in external quantum efficiency (EQE) at temperatures below 150 K, attributed to fluorescence trapping as confirmed by Monte Carlo ray‐tracing simulations. However, a comprehensive understanding of the underlying physical mechanisms and, more importantly, a general strategy to overcome them, has remained elusive.

Here, we experimentally identify two key factors impeding further cooling below cryogenic temperature: 1) a geometric symmetry‐related degradation of EQE at low temperatures, and 2) parasitic heating induced by water vapor condensation. By strategically reducing the crystal symmetry of 7.5% Yb^3+^:LLF, we experimentally demonstrate an increase in EQE with decreasing temperature, agreeing with the results from Monte Carlo fluorescence ray tracing simulation. Furthermore, our experimental and simulation results reveal that water vapor condensation effects become the dominant heating mechanism at low temperatures. More than just diagnosing these problems, our study establishes a dual‐path strategy of encompassing both material geometry and vacuum environment engineering, which provides a clear and universal roadmap for overcoming the current performance plateau. These insights deliver the critical design rules necessary to bridge the gap between theoretical promise and experimental achievement, paving a concrete pathway toward realizing efficient optical refrigeration at and beyond liquid‐nitrogen temperatures, from bulk cryocoolers to the emerging frontier of quantum optomechanics.

## 2 Results

### 2.1 Temperature‐Dependent Cooling Efficiency and the Role of External Quantum Efficiency

**FIGURE 1:**
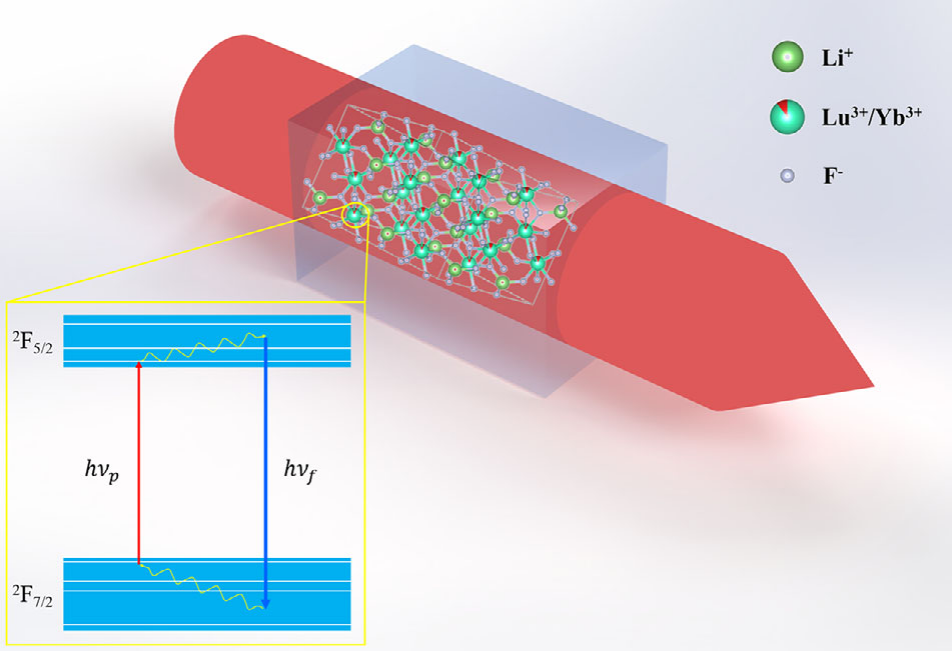
The schematic diagram of anti‐Stokes fluorescence cooling and energy‐level structure of Yb^3+^ in LLF. Doped Yb^3+^ ions substitute for Lu^3+^ ions sites in the host lattice. Insets show the cooling cycle within the Yb^3+^ ions.

To identify the key parameters limiting cryogenic cooling performance, we first establish the theoretical framework for optical refrigeration. The fundamental principle of anti‐Stokes fluorescence cooling (optical cooling), illustrated schematically in Figure 1, involves the absorption of coherent light at wavelength *λ*_p_, followed by fluorescence emission at a shorter wavelength *λ*_f_. This cooling mechanism relies on highly efficient fluorescence that removes thermal energy from the material. For rare‐earth‐doped hosts—such as the Yb^3+^: LLF crystal studied here—cooling occurs via transitions between the ^2^F_7/2_ (ground) and ^2^F_5/2_ (excited) manifolds of Yb^3+^ ions. Crystal‐field splitting resolves these manifolds into four and three Stark sublevels, respectively [24, 25, 50]. Under quasi‐equilibrium conditions (governed by Boltzmann statistics), pump photons excite electrons from the highest sublevel of ^2^F_7/2_ to the lowest sublevel of ^2^F_5/2_​. Subsequent thermalization via phonon absorption (nanosecond timescale) populates higher excited sublevels. Electrons then radiatively decay (millisecond timescale) to the ground manifold, emitting anti‐Stokes fluorescence photons with energy exceeding the absorbed pump photons, thereby extracting heat [2].

The cooling efficiency η_*c*_(λ_p_, *T*) follows the model of Sheik‐Bahae and Epstein [2, 51]:

(1) $$\eta_{c}\left(\lambda_{p},\;T\right)=\eta_{ext}\left[\frac{\alpha_{r}\left(\lambda_{p},\;T\right)}{\alpha_{r}\left(\lambda_{p},\;T\right)+\alpha_{b}}\right]\frac{\lambda_{p}}{\lambda_{f}\left(T\right)}-1$$

Here, η_*ext*_ is the external quantum efficiency. It is defined as: η_ext_ = η_esc_ *W_r_* /(η_esc_ *W*_r_  + *W*_nr_), and characterizes the probability that a photoexcited emitter generates a fluorescence photon escaping to free space from the system qualified by fluorescence escape efficiency *η*_esc_ relating to the effect of fluorescence re‐absorption and the total internal reflection in the host [52, 53]. *W*_r_ and *W*_nr_ denote radiative and nonradiative decay rates, respectively. The absorption efficiency is defined as: η_abs_(λ_p_, *T*) = α_r_(λ_p_, *T*)/(α_r_(λ_p_, *T*) + α_b_), which quantifies the fraction of pump photons participating in the cooling cycle. Here, α_r_(λ_p_, *T*) represents the resonant absorption coefficient and *α*_b_ corresponds to parasitic background absorption often coming from the transition metal ions. The resonant absorption coefficient α_r_(λ_p_, *T*) is temperature‐dependent, and follows the relation $\alpha_{r}\left(\lambda_{p},\;T\right)\propto 1/\left(1+\exp(\delta E_{g}/k_{B}T)\right)$ [53, 54], where *k_B_* is the Boltzmann constant and *T* is the sample temperature. δ*E*_g_ is the width of the ground‐state manifold. The average fluorescence wavelength λ_f_(*T*)∝1/ν_f_ (*T*) redshifts with decreasing temperature *h*ν_*f*_ (T)  ∼  *h*ν_f_ (0)  +  δ*E_u_*/(1 +  exp (δ*E_u_*/*k*_B_ *T*)), with δ*E_u_* representing the width of the excited‐state manifold [53].

The weak crystal‐field splitting in LLF broadens the rare‐earth ion manifolds, enhancing phonon coupling critical for efficient cooling [9, 25]. While optical cooling in Yb^3+^‐doped LLF and YLF has achieved cryogenic temperatures, cooling performance plateaus near LN_2_ temperatures. Although α_*r*_, λ_*f*_, and α_*b*_have been characterized down to 50 K, the influence of η_*ext*_ on cooling in this regime remains underexplored. We partition the nonradiative decay rate as *W_nr_* = *W_nr1_* + *W_nr0_*, where *W_nr1_*, *W_nr1_* ​ arises from reabsorption‐induced nonradiative decay, and *W_nr0_* = *W_mp_* +Σ*iW_i_* includes multiphonon decay *W_mp_* and other channels (e.g., cooperative processes) [52, 55]. Thus, η_*ext*_ simplifies to: η_*ext*_ = η_*q*_η_*esc*_, where η_*q*_ = *W_r_* /(*W_r_*  + *W*_*nr*0_). Here, η_*q*_ is called internal quantum efficiency, and η_*esc*_ named the fluorescence escape rate can be determined via Monte Carlo ray‐tracing simulations [56].

### 2.2 Geometric Symmetry and Fluorescence Escape Efficiency

**FIGURE 2:**
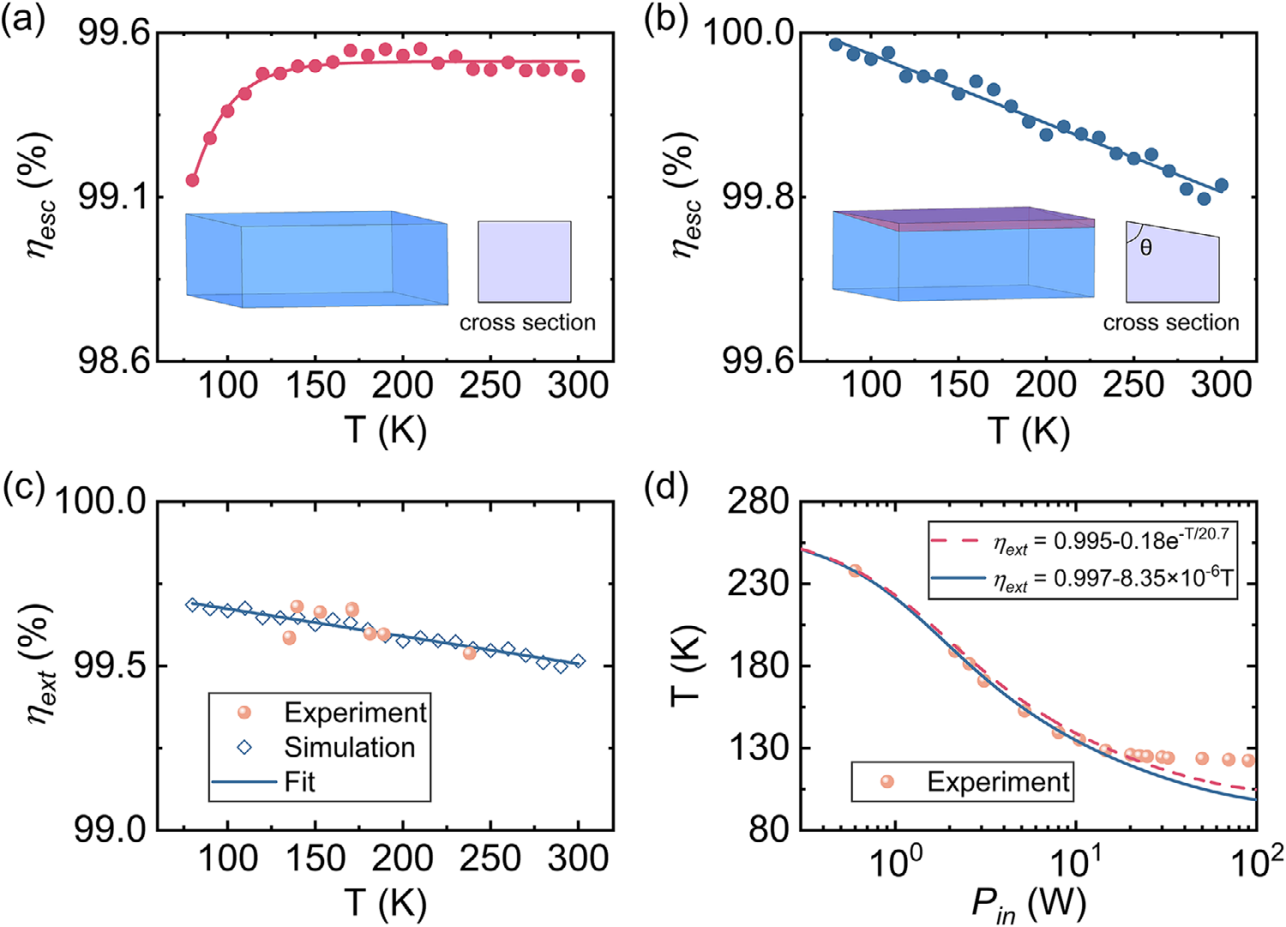
Temperature‐dependent performance of 7.5% Yb^3+^:LLF crystals. (a) Simulated escape efficiency *η*_esc_ for a Brewster‐angle‐cut sample with a square cross‐section, assuming constant quantum efficiency *η_q_* at room temperature. The inset shows the geometry and cross section of the sample. (b) Simulated *η*_esc_ for a Brewster‐angle‐cut sample with corner angle θ = 85° over 80 K to 300 K. The inset shows the geometry and cross section of the sample. (c) External quantum efficiency for Brewster‐angle‐cut sample with corner angle θ = 85°: experimental data (red solid spheres) and simulation (hollow squares). The simulation is fitted by *η*_ext_ = 0.997–8.35 × 10^−6^ *T* (solid line). (d) Measured steady‐state sample temperature (solid dots) vs. pump power and model fits: temperature‐dependent *η*_ext_ for a square cross‐section Brewster sample (dashed line) and for the θ = 85° Brewster sample (solid line).

Figure 2a presents the simulated fluorescence escape efficiency *η_esc_* for a 7.5%Yb^3+^:LLF sample featuring a Brewster angle end face and a square cross‐section, calculated via Monte Carlo fluorescence ray tracing over the temperature range of 80 to 300 K. As the lattice temperature decreases, reabsorption diminishes, leading to increased trapping of fluorescence due to total internal reflection. Notably, in the cryogenic regime, this trapped fluorescence is inevitably absorbed by impurities. This occurs because the significant reduction in reabsorption at low temperatures suppresses the random re‐emission pathways. These pathways normally provide opportunities for trapped fluorescence to escape, but this ‘recycling’ mechanism becomes ineffective. Figure 2b shows *η_esc_* for a Brewster‐angle‐cut sample with a square cross‐section (corner angle θ = 85°) of 7.5%Yb^3+^:LLF, over temperature range of 80 to 300 K. The simulations reveal that *η_esc_* increases progressively as temperature drops, exceeding 99.9% near 130 K and approaching unity at 80 K. The specific corner angle of 85° was selected as a practical optimum, balancing performance gain with fabrication feasibility. Parametric simulations indicated that while reducing the angle from 90° improves the fluorescence escape efficiency *η_esc_*, the benefit saturates near 85°. This design provides a substantial enhancement in *η_esc_* while maintaining mechanical integrity and preserving a large optical aperture for future integration with multi‐pass pump systems (e.g., Herriott cell), which is crucial for achieving high absorption at cryogenic temperatures.

The simulated external quantum efficiency *η_ext_* for the Brewster‐cut sample (3.1 × 3.1(2.8) × 10.0 mm^3^, with corner angle of θ = 85°), assuming constant internal quantum efficiency *η_q_*, is shown in Figure 2c. *η_ext_* increases linearly with decreasing temperature, following the relation *η_ext_* = 0.997–8.35 × 10^−6^ *T*, driven primarily by the rise in *η*_esc_. Experimental *η*_ext_ values, derived from laser cooling measurements under varying pump power using the equilibrium thermodynamic equation *CdT*/*dt* = *η_c_P_in_* (1‐exp(‐2*α_r_* (*T*)*L*)) + *P*_rad_, where *C* is heat capacity, *L* is sample length, and *P*_rad_ is the blackbody radiation heat load, exhibit excellent agreement with the simulated linear trend. This confirms that *η_ext_* increases for this geometry as lattice temperature decreases, enhancing laser cooling performance.

Figure 2d plots the measured steady‐state temperatures of the 7.5%Yb^3+^:LLF sample pumped at 1020 nm against incident power of laser. The sample was cooled to ∼135 K with 10 W of laser power. The experimental data align closely with the theoretical fitting curve (solid line) based on the equilibrium thermodynamic equation incorporating the linearly fitted *η_ext_* from Figure 2c. For comparison, the dashed line represents the theoretical prediction using a *η_ext_* = 0.995–0.18exp(‐*T*/20.7) of a sample featuring a Brewster angle end face and a square cross‐section. The experimental temperatures fall below this theoretical prediction line, demonstrating the increase in EQE with cooling. However, below ∼135.0 K, the measured temperature deviates upward from the prediction based on the linearly increasing *η_ext_*. For instance, at 90 W pump power, the sample cooled only to 122.5 K, significantly higher than the predicted 100 K. This deviation is attributed to water vapor condensation on the sample surface and supporting fibers at low temperatures.

### 2.3 Parasitic Heating from Water Vapor Condensation

According to Arden Buck equation [57], the saturation vapor pressure of water at ∼140 K falls within the range of 10^−6^∼10^−7^ Pa. The water molecules contribute a pressure percentage of ∼1 × 10^−6^ Pa when the vacuum chamber is pumped to ∼3 × 10^−6^ Pa. Therefore, water vapor condensation starts to take an increasingly important role as the sample is being cooled below ∼140 K. The condensed water molecules on the sample surfaces can absorb fluorescence light and cause an extra heat load, denoted as *P_extra_* ​.

**FIGURE 3:**
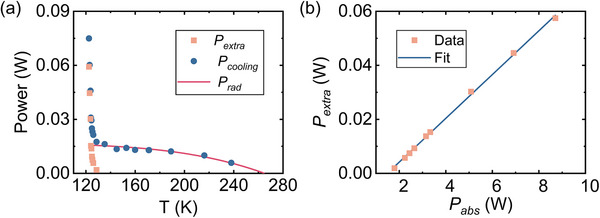
Experimental characterization of cooling power and parasitic heat load. (a) Measured cooling power of the 7.5% Yb^3+^:LLF sample versus steady‐state temperature. Dots represent the cooling power. Squares represent the extra heat load (excluding the calculated blackbody radiation load). The red line shows the calculated blackbody radiation load on the sample. (b) Extra heat load versus absorbed pump laser power. The solid line is a linear fit.

During the process of laser cooling, the steady‐state temperature of the sample is achieved when the laser cooling power balances out the heat load on the sample. The laser cooling power can be determined in the experiment from the expression *P_cooling_* = *η_c_P_in_* (1‐exp(‐2*α_r_* (*T*)*L*)). The solid circles in Figure 3a show the variation of *P_cooling_* with the steady‐state temperature under various pump laser powers for the 7.5% Yb^3+^:LLF sample. The blackbody radiation from the surrounding environment, is given by $P_{rad}=\frac{\varepsilon_{s}A_{s}\sigma }{1+\chi }\left(T_{c}^{4}-T^{4}\right)$, is a major contributor to the heat load during laser cooling. Here, ε and A represent the emissivity and surface area, respectively. And the subscripts *s* and *c* refer to the sample and its surrounding clamshell, respectively. The Stefan–Boltzmann constant σ is given as 5.67 × 10^−8^ Wm^−2^K^−4^. The parameter χ is defined as $\chi =\frac{\left(1-\varepsilon_{c}\right)\varepsilon_{s}A_{s}}{\varepsilon_{c}A_{c}}$. The red line in Figure 3a presents the calculated values of *P_rad_* for various steady‐state temperatures. When the steady‐state temperature is lower than 135 K, the aforementioned extra heat load *P_extra_* is speculated to play an important role. *P_extra_* is determined by subtracting the value of *P_rad_* from that of *P_cooling_*, as shown by the solid orange squares in Figure 3a. Since *P_extra_* ​ is ascribed to the absorption of fluorescence light by condensed water vapor, its magnitude should be proportional to the absorbed pump laser power *P_ab_*_s_ = *P_in_* (1‐exp(‐2*α_r_* (*T*)*L*)). Figure 3b presents the correspondence between *P_extra_* and *P_abs_* as the steady‐state temperature decreases from ∼135.0 to ∼122.5 K. The solid line represents a linear fit to the experimental data. The excellent agreement confirms our analysis and identifies the origin of the parasitic heat load. The zero value of *P_extra_* is obtained when *P_abs_* is equal to 1.5 ± 0.02 W, corresponding to a steady‐state temperature of 136.9±2 K. This value, within uncertainty, align with the aforementioned onset temperature of ∼135.0 K, consistently indicating the threshold for noticeable heating.

**FIGURE 4:**
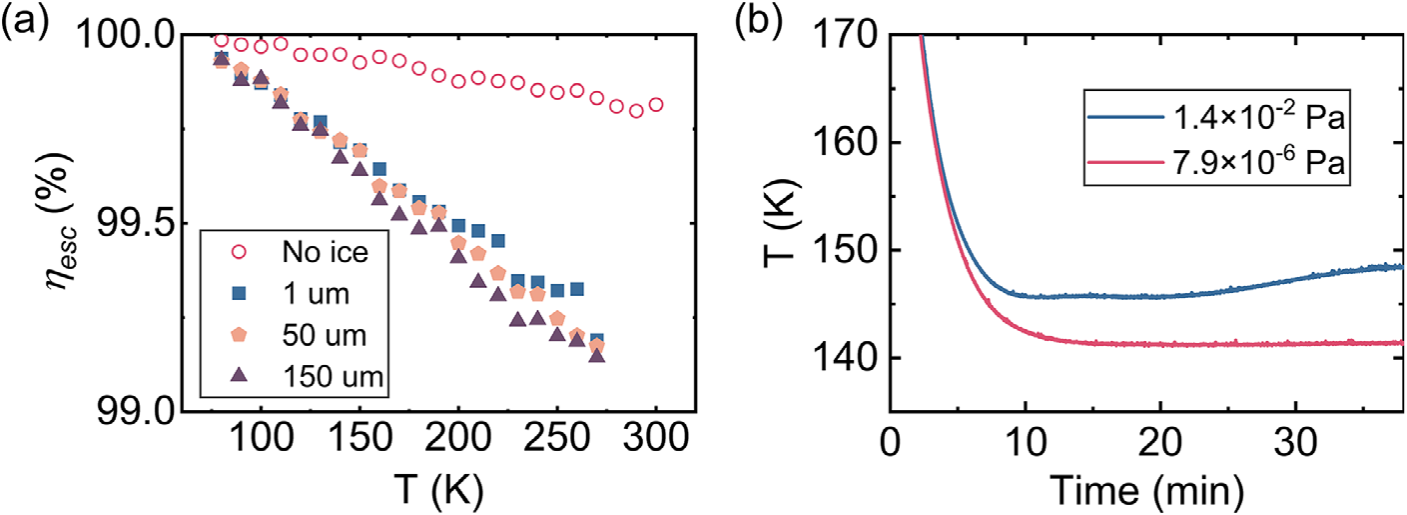
Theoretical analysis of fluorescence escape efficiency and its impact on cooling performance. (a) Fluorescence escape efficiency *η_esc_* as a function of temperature for a 7.5% Yb^3+^:LLF sample with varying ice film thickness. (b) Resulting cooling temperature of the same sample under an identical pump power but at two different vacuum levels: 1.4 × 10^−^^2^ Pa and 7.9 × 10^−^^6^ Pa. The presented values are mean ± SD.

To evaluate the effect of surface condensation, we performed Monte Carlo ray‐tracing simulations of fluorescence propagation in crystals coated with an ice layer of varying thicknesses (80–270 K), using the optical constants of ice (*n* = 1.33, *α* = 0.26 cm^−1^), which are representative of amorphous ice in the near‐infrared region [58, 59, 60]. The use of constant values across the simulated temperature range is justified, as the temperature dependence of these optical constants within the 80–270 K window is negligible for the purpose of our comparative analysis of parasitic heating [58, 59, 60]. Figure 4a shows that the fluorescence escape efficiency *η_esc_* decreases with ice coating compared to a bare crystal. This arises from a competing effect: although the ice film reduces internal reflection, promoting fluorescence escape into the ice, the higher absorption of ice leads to substantial losses. This is particularly severe for rays at high grazing angles, which exhibit extended path lengths in the ice. The strength of this inhibitory effect increases with temperature. At lower temperatures, the resonant absorption coefficient of the crystal itself drops significantly, reducing reabsorption and re‐emission events. Consequently, a smaller proportion of fluorescence is scattered into oblique angles that result in long path lengths through the ice. The average path length in the ice is therefore shorter, diminishing the impact of the ice film. The extra heat load from ice absorption, denoted as $P_{extra}^{ice}$ ​, is the sum of the absorbed pump power and fluorescence power in the ice layer: $P_{extra}^{ice}=P_{abs,ice}^{flu}+P_{abs,ice}^{pump}=\left(\eta_{esc}^{no\;ice}-\eta_{esc}^{ice}\right)P_{flu}+\left(2P_{in}-P_{abs}\right)\left(1-e^{-\alpha_{ice}l_{ice}}\right)$, where $\eta_{esc}^{no\;ice}$ and $\eta_{esc}^{ice}$ represent the fluorescence escape efficiencies for samples without and with ice, *P_flu_* = *P_cooling_* + *P_abs_* ​, is the total fluorescence power, α_*ice*_ is the absorption coefficient of ice, *l_ice_* is the ice thickness, *P_in_* ​ is the incident pump power, and *P_abs_* is the power absorbed by the crystal. The calculated $P_{extra}^{ice}$ linearly scaling with the absorbed pump laser power. To more clearly elucidate the effect of condensation, the sample's temperatures were measured under different pressure conditions at a fixed pump power 18.5 W (Figure 4b). At a higher pressure (1.4 × 10^−2^ Pa), where condensation occurs, the temperature stabilized at 148.3 ± 0.7 K. In contrast, at a much lower pressure (7.9 × 10^−6^ Pa), where condensation is negligible, a temperature of 141.3 ± 0.7 K was reached. After accounting for convective heat loads, the theoretical expected temperature at 1.4 × 10^−2^ Pa is computed to be 142.3 ± 0.7 K. The 6.0 K discrepancy substantially exceeds the measurement uncertainty (±0.7 K), providing robust, direct evidence that $P_{extra}^{ice}$ is the dominant parasitic heat load.

### 2.4 Cooling Potential with Optimized Geometry

**FIGURE 5:**
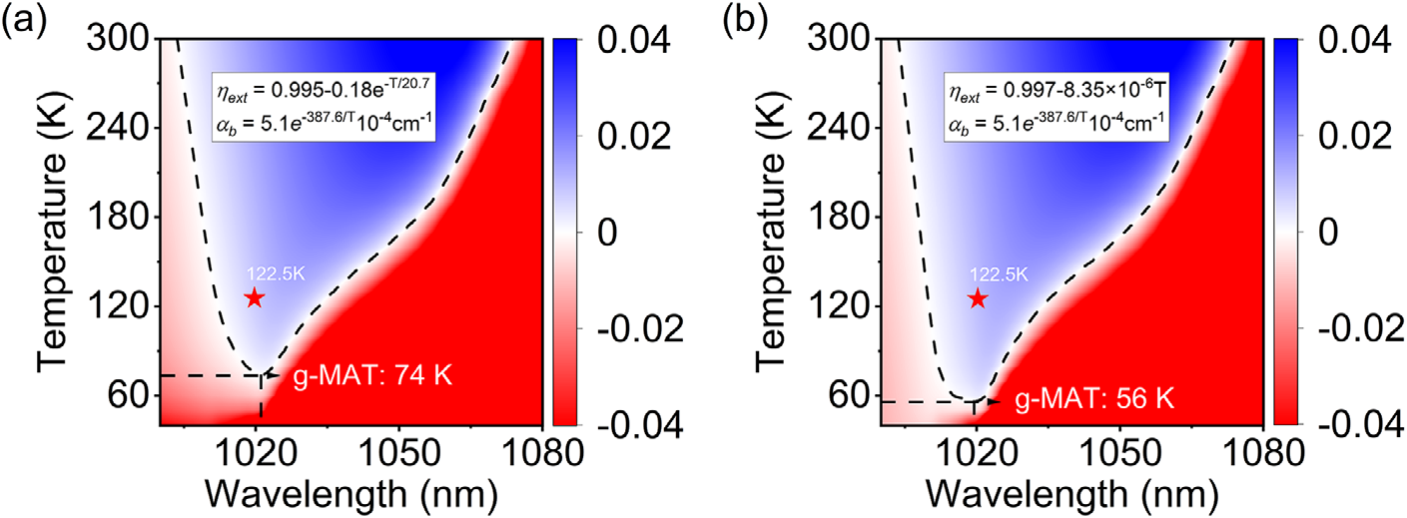
Cooling window of 7.5%Yb:LLF sample with different geometries. (a) A Brewster‐angle‐cut sample with a square cross‐section: *η_ext_* = 0.995–0.18exp(‐*T*/20.7). Predicted g‐MAT = 74 K. (b) Brewster‐angle‐cut sample with a square cross‐section (corner angle θ = 85°): *η_ext_* = 0.997‐8.35 × 10^−6^ *T*. Predicted g‐MAT = 56 K. Predictions assume the temperature‐dependent background absorption reduction reported in Refs. [8, 9]. The solid star marks the minimum temperature (122.5 K) achieved experimentally in this work, highlighting the performance gap attributable to residual parasitic heat loads (e.g., condensation) and limited pump absorption in the current setup.

Achieving cryogenic temperatures via laser cooling critically depends on minimizing parasitic background absorption *α_b_*. Recent results for YLF: 5%Yb^3+^‐0.0016%Tm^3+^ crystals demonstrate that *α_b_* decreases by an order of magnitude, from ∼10^−4^ to ∼10^−5^ cm^−1^, as the temperature drops from 300 to 100 K [8]. A similar temperature dependence of *α_b_* is observed in Yb^3+^:LLF crystals, enabling their cooling below liquid nitrogen (LN_2_) temperatures. For our sample, the background absorption coefficient at room temperature is *α_b_* ​(300 K) = 1.4 × 10^−4^ cm^−1^, consistent with the purity achievable in state‐of‐the‐art ultra‐pure fluoride crystal growth [8, 9]. Its temperature dependence follows *α_b_* (*T*) = 5.1 exp(−387.6/*T*) ×10^−4^ cm^−1^, as reported in refs. [8, 9]. It should be noted that while the trend of sharply decreasing background absorption with temperature is well‐established for ultra‐pure fluoride hosts, the precise empirical fit at the lowest temperatures introduces some uncertainty in the absolute predicted g‐MAT values. Nevertheless, this uncertainty does not affect the qualitative conclusion that breaking geometric symmetry substantially lowers the g‐MAT by improving *η_ext_*. Figure 5a,b present the simulated g‐MAT for the 7.5% Yb^3+^:LLF sample. The simulations utilize an external quantum efficiency *η_ext_* modeled as *η_ext_* = 0.995–0.18exp(‐*T*/20.7) for a Brewster‐angle end‐face sample and *η_ext_* = 0.997‐8.35 × 10^−6^ *T* for a sample with a square cross‐section and corner angle θ = 85°. Crucially, the simulations for the θ = 85° configuration (representing the current sample geometry) predict a g‐MAT of 56 K, far below the LN_2_ temperature. This reveals that for the same sample, upon introducing an 85° cut angle, the detrimental effect of fluorescence trapping on laser cooling is remarkably mitigated at low temperatures. As a result, the g‐MAT decreases from 74 to 56 K. Despite successfully mitigating the impact of fluorescence trapping during the experiment, the lowest temperature achieved was 122.5 K marked in Figure 5a,b, still showing a substantial gap compared to the sample's g‐MAT of 56 K. The primary limitations are the additional thermal load from condensation and the low pump laser absorption by the sample. In the current experiment, a double‐pass geometry has been adopted and the cooling laser beam only passes the crystal sample two times, the absorption rate was merely 8.7% at 122.5 K. Implementing a multi‐pass geometry such as a Herriott cavity [54] would dramatically enhance the absorbed power, particularly at cryogenic temperatures where the resonant absorption coefficient is low. For instance, increasing the number of passes from 2 (current double‐pass) to 150 would raise the absorption efficiency at 100 K from ∼3% to ∼89%, corresponding to a ∼30‐fold increase in net cooling power. This could lower the achievable temperature for our sample from ∼122.5 K to approximately 65 K, closely approaching its predicted g‐MAT. In the next stage of work, we will significantly address this issue by enhancing the absorption through a Herriott cavity [54].

However, surface condensation poses a significant challenge for deep cryogenic laser cooling. It introduces an additional heat load *P_extra_* ​, hindering further temperature reduction and potentially preventing cooling down to LN_2_ temperatures. This limitation was experimentally observed in our previous work, where cooling stalled around 135.0 K, and is consistent with findings in the Yb^3+^:YLF crystal by the M. Sheik‐Bahae group [61, 62]. In the resonant intra‐cavity enhancement absorption scheme for laser cooling in a 10^−3^ Pa vacuum, lasing stops when the sample, cooled by the laser to 168 K, experiences the condensation effect [61]. To overcome this barrier and achieve sub‐LN_2_ cooling, stringent mitigation strategies are essential. These include prolonged baking of the vacuum chamber and internal components to minimize residual gas, and the use of a cryogenic pump to pre‐condense residual gas onto its cold head, thereby diminishing condensation on the crystal surface itself.

## 3 Discussion and Conclusions

The pursuit of deeper laser cooling reveals a fundamental shift in the dominant limiting factors with temperature. At or near room temperature, performance is primarily constrained by parasitic background absorption (*α_b_*) from impurities and by the internal quantum defect (1‐*η_q_*), which encompasses non‐radiative channels such as multiphonon relaxation. However, as the temperature drops below ∼150 K, these channels are exponentially suppressed. In this critical cryogenic regime, our work identifies and addresses the two emergent, dominant bottlenecks: geometry‐dependent fluorescence trapping (governing *η_esc_*) and condensation‐induced parasitic heating. This clarifies the long‐standing discrepancy between theoretical predictions and experimental plateaus and redefines the optimization priorities for sub‐100 K optical refrigeration.

We have experimentally investigated two fundamental factors hindering laser cooling of solids toward liquid nitrogen temperatures: geometric symmetry‐related fluorescence trapping and water vapor condensation parasitic heating. Laser cooling of a Brewster‐cut 7.5% Yb^3+^:LLF crystal with reduced geometric symmetry (corner angle θ = 85°) has shown that the fluorescence escape efficiency *η_esc_* can be effectively increased compared to the case without symmetry reduction (corner angle θ = 90°). This increase of *η_esc_* becomes more pronounced and critical when the sample is being cooled to low temperatures. The external quantum efficiency *η_ext_* is improved accordingly and leads to a decreased sample g‐MAT from 74 to 56 K. Meanwhile, we have also identified water vapor condensation at sample surfaces as the dominant parasitic heat load when it is being cooled below 135 K (chamber vacuum ∼10^−6^ Pa), which directly causes the persistent discrepancy between theoretical limits and experimental performance. Our experimental minimum of 122.5 K, marked in Figure 5a,b, highlights the current gap attributable to these residual loads. Thus, our work establishes an experimentally validated dual‐path strategy for deeper cooling: geometric symmetry optimization to boost fluorescence escape, and stringent vacuum control to mitigate condensation‐induced heating.

Beyond achieving lower temperatures in bulk solids, our findings provide critical insights for adjacent frontier fields. The principles of managing fluorescence escape and parasitic heating are universally applicable. For instance, the limitation of fluorescence trapping is not unique to Yb^3+^:LLF but is a general challenge for many rare‐earth‐doped solids (e.g., Yb^3+^:YLF, Tm^3+^‐doped systems). Our geometry‐dependent Monte Carlo model thus serves as a universal tool for optimizing the external quantum efficiency across different host materials. Moreover, the same principles and computational toolkit can be directly applied to the design of micro‐ and nano‐particles for emerging applications in levitated optomechanics. Strategic shape engineering, such as employing irregular nanoparticles, nanorods, or core‐shell structures with broken symmetry, can be tailored to maximize *η_esc_* and thus enhance cooling efficiency. Our model provides a predictive design platform for such particle geometries.

Building upon this design paradigm, our work bridges the gap between macroscopic laser refrigeration and mesoscopic quantum optomechanics. Recent breakthroughs in cooling the motional degrees of levitated nanoparticles to the quantum ground state [63] have typically neglected the internal thermodynamic temperature of the particle itself, which remains elevated due to laser absorption [12]. This high internal temperature constrains pump power and introduces thermal decoherence, limiting the preparation of macroscopic quantum states [16]. Our strategies offer a clear pathway to overcome this bottleneck: The geometric symmetry reduction strategy can be directly applied to the design of optically levitated micro‐ and nano‐particles, maximizing fluorescence heat removal and enabling higher pump power without thermal damage. This approach is particularly powerful when the particles are composed of high‐quality anti‐Stokes cooling materials (e.g., ultra‐pure rare‐earth‐doped nanocrystals and nano semiconductors). Here, a crucial distinction arises compared to conventional Doppler cooling of solids. In the latter, parasitic heating often stems from the direct absorption of the pump laser by the host. In contrast, for particles made of ideal anti‐Stokes materials, the dominant low‐temperature loss becomes the fluorescence trapping and subsequent impurity absorption we have identified a radiative loss channel that our geometric design directly mitigates. Therefore, leveraging such materials within a Doppler cooling framework creates a synergistic advantage: the particle's internal temperature is actively managed via efficient anti‐Stokes fluorescence, while its motion is cooled via the Doppler interaction. This hybrid cooling paradigm, enabled by materials and geometry co‐optimized using principles from our work, could significantly advance the coherence times and stability of levitated quantum systems. Furthermore, our quantification of condensation‐induced parasitic heating highlights a crucial, often overlooked heat source in ultra‐high vacuum systems, which is vital for experiments aiming to co‐cool both the internal and motional degrees of freedom of a levitated particle to the quantum regime.

In conclusion, our study not only resolves a long‐standing performance gap in solid‐state optical refrigeration by providing a concrete dual‐path strategy but also delivers a set of critical design rules that are broadly applicable from engineering bulk crystals for space‐borne cryocoolers to optimizing micro‐structures for on‐chip quantum technologies. By identifying and providing solutions to these fundamental limitations, this work establishes a foundation for advancing optical refrigeration toward liquid‐nitrogen temperatures and opens avenues for exploring ultralow thermodynamic temperatures in levitated systems, thereby paving the way for practical optical cryocoolers and advancing the broader pursuit of macroscopic quantum phenomena.

## 4 Experimental Section

### 4.1 Experimental Setup for Spectroscopic Characterization

**FIGURE 6:**
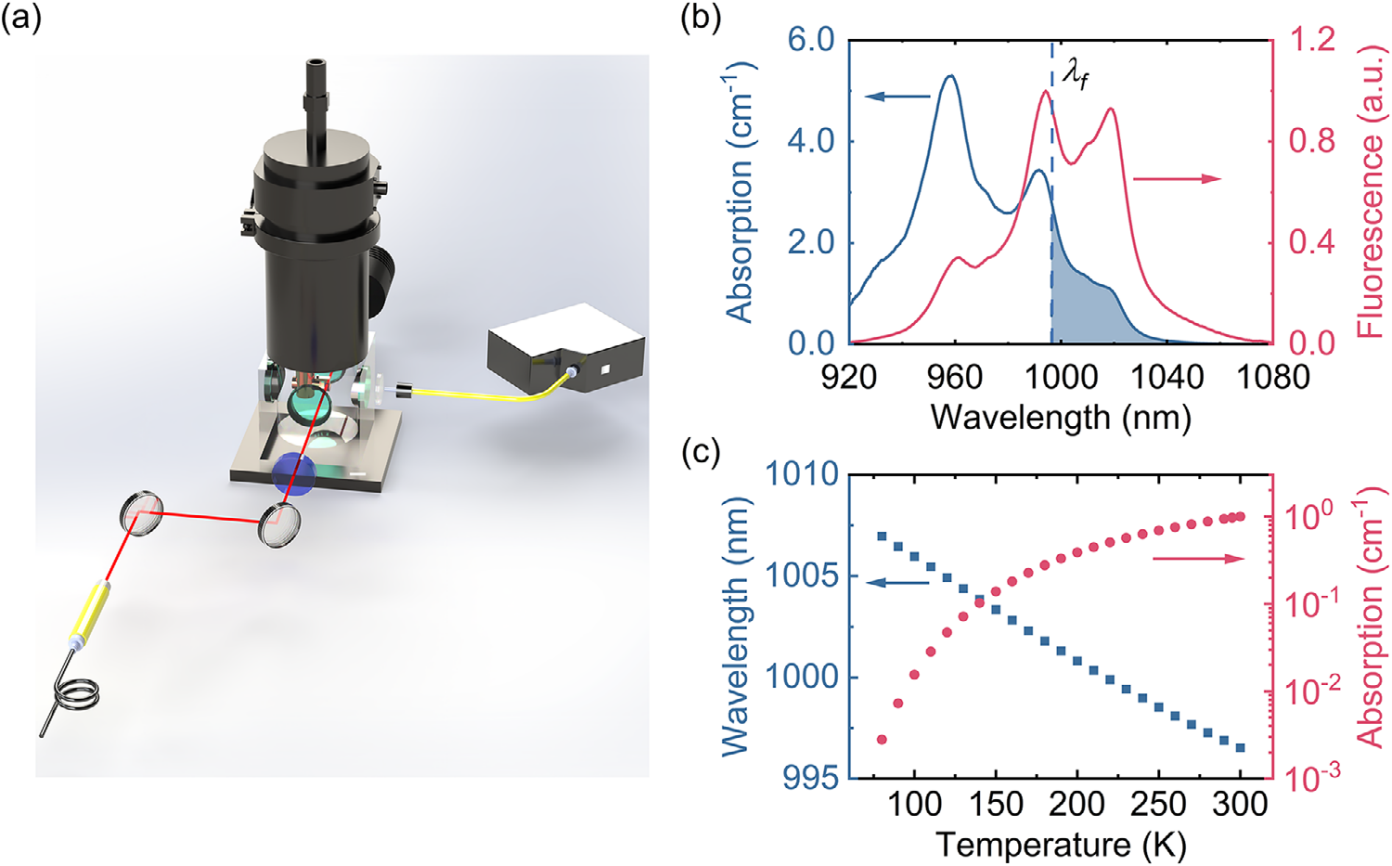
Experimental setup and spectroscopic characterization of Yb^3+^:LLF crystal. (a) Schematic of the experimental setup for fluorescence and absorption spectroscopy, covering temperatures from 80 to 300 K, including the average fluorescence wavelength *λ_f_*. (b) Absorption and fluorescence emission spectra of the 7.5% Yb^3+^: LLF crystal at 300 K. The shaded region indicates the “cooling tail” (*λ_p_* > *λ_f_*) corresponding to the absorption spectrum. (c) Temperature dependence of the measured average fluorescence wavelength *λ_f_* and the resonant absorption coefficient at 1020 nm.

Figure 6a illustrates the experimental setup for measuring polarization‐resolved fluorescence spectra across a temperature range of 80–300 K at 10 K intervals. A 2 mW diode laser (*λ* = 914 nm) pumps a 2 × 2 × 5 mm^3^ sample. The laser beam passes through the crystal center, with its a‐axis oriented toward the fluorescence collection window. The crystal is clamped onto a copper cold‐finger within a high‐vacuum cryostat (Janis VPF‐100), using indium foil to ensure thermal contact. A polarizer preceding a 600‐µm‐diameter collection fiber enables acquisition of polarization‐resolved fluorescence spectra (E∥c [π] and E⊥c [σ]) at varied temperatures. The fluorescence collection system, which included a spectrometer (Ocean Optics Maya Pro 2000NIR), was calibrated using a standard tungsten halogen lamp (Ocean Optics). Temperature‐dependent absorption spectra were derived via the reciprocity theorem and Füchtbauer‐Ladenburg equation [64]:$\;\alpha \left(\lambda ,\;T\right)\propto \lambda^{5}S\left(\lambda ,T\right)e^{hc/\lambda k_{B}T}$, where *S(λ,T)* is the measured fluorescence spectral density at temperature T. Absorption spectra were calibrated using absorption coefficients of 1020 nm determined via the Beer‐Lambert law.

Figure 6b presents the fluorescence spectra *S*(λ,  *T*) for Yb^3+^:LLF in E∥c orientations, alongside the resonant absorption spectrum α_*r*_(λ, *T*) in E∥c obtained via reciprocity at 300 K. The pump laser wavelength resides in the “cooling tail” (shaded region in Figure 6b), a spectral region below the mean fluorescence wavelength *λ_f_* ​. The mean fluorescence wavelength was calculated as: $\lambda_{f}\left(T\right)=\int \lambda S\left(\lambda ,\;T\right)d\lambda /\int S\left(\lambda ,\;T\right)d\lambda$, accounting for polarization weights:$\;\lambda_{f}\left(T\right)=\frac{1}{3}\lambda_{f}^{\pi }\left(T\right)+\frac{2}{3}\lambda_{f}^{\sigma }\left(T\right)$ [33]. Figure 6c depicts *λ_f_* ​(*T*) and resonant absorption coefficients *α_r_* ​(*T*) for 7.5% Yb^3+^:LLF between 80 and 300 K. As temperature decreases, *λ_f_* ​ redshifts due to Boltzmann‐driven repopulation of lower‐energy excited states. Concurrently, *α_r_* ​ decreases by nearly three orders of magnitude from 300 to 80 K.

### 4.2 Vacuum System and Pressure Control for Condensation Studies

A detailed description of the vacuum environment is critical for the low‐temperature experiments, especially for quantifying condensation‐induced parasitic heating. The main vacuum chamber was constructed of stainless steel and was baked at approximately 75°C for more than 48 h prior to the experiments to minimize outgassing, particularly of water vapor. The base pressure was achieved using a turbomolecular pump (Agilent TwisTorr 84 FS) directly mounted on the chamber and backed by a dry scroll pump (Agilent IDP‐10). A manual right‐angle valve was installed between the turbomolecular pump and the backing pump. A liquid‐nitrogen‐cooled cold trap was mounted on the top flange of the main chamber to cryogenically adsorb residual water vapor during cooldown. The pressure was monitored using an ionization gauge. To experimentally study the impact of water vapor condensation, a controlled reduction of the vacuum level was necessary. This was achieved by isolating the chamber from the dry scroll pump using the aforementioned angle valve. To establish the “high‐pressure” condition (as reported in Figure 4b), the following procedure was used after the system had reached its base pressure: 1) the angle valve was closed, isolating the backing dry scroll pump from the chamber; 2) the turbomolecular pump was then switched off, while the backing dry scroll pump remained in operation. This procedure allowed the chamber pressure to rise in a controlled manner from the base pressure of ∼7.9 × 10^−6^ Pa to the target pressure of ∼1.4 × 10^−2^ Pa, primarily through the gradual release of adsorbed water vapor from the chamber walls and other optical components. The pressure was allowed to stabilize before cooling experiments commenced at this elevated pressure.

### 4.3 Laser Cooling Efficiency Measurement

The cooling efficiency *η_c_* ​(*λ*,*T*) at room temperature was measured using Laser‐Induced Temperature Modulation Spectroscopy (LITMoS) test [65] under vacuum (10^−4^ Pa): η_*c*_(λ, *T*) =  Δ*T*/*KP_abs_*, where Δ*T* is the laser‐induced temperature change, *P_a_* ​*_bs_* is the absorbed power, and *K* is a scaling constant inversely proportional to the thermal load. The external quantum efficiency *η_ext_* ​ and background absorption coefficient *α_b_* ​, key determinants of laser cooling performance, were quantified by fitting wavelength‐dependent *η_c_* ​(*λ*,*T*) measurements to Equation (1) using least‐squares regression. Experimental details are provided in Ref. [65].

### 4.4 Laser Cooling Experimental Set‐Up

For laser cooling, a tunable fiber laser (Precilasers Co., Ltd., FL‐NSF‐1080‐80‐CW) pumped an optically polished, Brewster‐angle‐cut 7.5% Yb^3+^:LLF crystal. The crystal was suspended by two 100‐µm‐diameter fibers within a nanoblack‐coated copper clamshell with high absorptivity of 900–1080 nm and low thermal emissivity. A double‐pass geometry with E∥c orientation maximized absorption. The clamshell temperature was maintained at ∼266 K using an anhydrous ethanol chiller within a vacuum chamber of P ≈ 3 × 10^−6^ Pa. Sample temperature was monitored in real‐time via differential luminescence thermometry. Additional details can be found in Ref. [9].

### 4.5 Monte Carlo Ray‐Tracing Simulations

Monte Carlo fluorescence ray‐tracing simulations were conducted to determine the fluorescence escape efficiency, *η_esc_*, for samples with various geometries and surface conditions (e.g., with or without ice coating). The optical constants of the Yb^3+^:LLF crystal and ice (*n* = 1.33, *α* = 0.26 cm^−1^) were incorporated into the simulations. A total of 10^5^ fluorescent rays were initialized with random spatial origin, propagation direction, polarization, and emission wavelength sampled from the fluorescence spectrum. Ray propagation was simulated in discrete spatial steps. Absorption was assessed at each step according to the Beer–Lambert law. Rays that were absorbed were either lost to background absorption or re‐emitted with updated properties. Upon reaching an interface, Fresnel reflection was evaluated. Reflected rays re‐entered the propagation cycle, whereas transmitted rays were registered as escaped in the absence of an ice layer. For ice‐coated samples, transmitted rays underwent further propagation through the ice with stepwise absorption checks; escape was counted only if transmission occurred through the ice without absorption. The process was iterated until all rays were either absorbed or had escaped. The simulation code, partially adapted from Ref. [66], is available from the corresponding authors upon reasonable request.

### 4.6 Statistical Analysis

The reproducibility of the laser cooling experiments was confirmed at least three times. Exponential fitting was used in Figure 2a, while linear fitting was used in Figure 2b,c. The absorbed power corresponding to the zero value of *P_extra_* in Figure 3b was determined by linear fitting (error derived from the fit). The stabilized temperatures in Figure 4b are presented as mean ± standard deviation (SD). The fluorescence spectrum in Figure 6b was normalized to its highest peak.

## Funding

The research is supported by the Project for Young Scientists in Basic Research under grant No. YSBR‐120; National Natural Science Foundation of China (11604100, 62175066, 12474404); Director's Fund of the Technical Institute of Physics and Chemistry, CAS (E2A9R40101); Key Research Program of the State Key Laboratory of Cryogenic Science and Technology, CAS.

## Conflicts of Interest

The authors declare no conflicts of interest.

## Data Availability

The data that support the findings of this study are available from the corresponding author upon reasonable request.
